# Influence of diabetes on microbiome in prostate tissues of patients with prostate cancer

**DOI:** 10.3389/fonc.2024.1445375

**Published:** 2024-08-16

**Authors:** Jin-Jae Lee, Jung Kwon Kim, Bumjo Oh, Sung Kyu Hong, Bong-Soo Kim

**Affiliations:** ^1^ Department of Life Science, Multidisciplinary Genome Institute, Hallym University, Chuncheon, Republic of Korea; ^2^ Department of Urology, Asan Medical Center, University of Ulsan College of Medicine, Seoul, Republic of Korea; ^3^ Deparment of Family Medicine, Seoul Metropolitan Government-Seoul National University Boramae Medical Center, Seoul, Republic of Korea; ^4^ Department of Urology, Seoul National University College of Medicine, Seoul, Republic of Korea; ^5^ Department of Urology, Seoul National University Bundang Hospital, Seongnam, Republic of Korea

**Keywords:** microbiome, prostate cancer, prostate tissue, diabetes, pathologic group

## Abstract

**Background:**

Although microbiota in prostatic tissues of patients with prostate cancer have been studied, results of different studies have been inconsistent. Different ethnicity of study subjects, different study designs, and potential contaminations during sample collection and experiments might have influenced microbiome results of prostatic tissues. In this study, we analyzed microbiota and their potential functions in benign and malignant tissues of prostate cancer considering possible contaminants and host variables.

**Materials and methods:**

A total of 118 tissue samples (59 benign tissues and 59 malignant tissues) obtained by robot‐assisted laparoscopic radical prostatectomy were analyzed and 64 negative controls (from sampling to sequencing processes) were included to reduce potential contaminants.

**Results:**

Alteration of the microbiome in prostate tissues was detected only in patients with diabetes. Furthermore, the influence of diabetes on microbiome was significant in malignant tissues. The microbiome in malignant tissues of patients with diabetes was influenced by pathologic stages. The relative abundance of *Cutibacterium* was reduced in the high pathologic group compared to that in the intermediate group. This reduction was related to microbial pathways increased in the high pathologic group.

**Conclusion:**

Results of this study indicate that diabetes can influence the progression of prostate cancer with microbiome alteration in prostate tissues. Although further studies are necessary to confirm findings of this study, this study can help us understand tissue microbiome in prostate cancer and improve clinical therapy strategies.

## 1 Introduction

Prostate cancer (PC) is the third most common cancer worldwide. Its incidence is steadily increasing in Asia (1). Risk factors for PC include chronic inflammation, microbial infections, and environmental factors such as diet and lifestyle, age, ethnicity, and family history (2, 3). These factors can lead to dysbiosis of the human microbiome, which plays a pivotal role in health and disease (4, 5). PC is a prime candidate for research due to the significant role of chronic inflammation as a risk factor. The proximity of the prostate to the urinary tract allows for potential exposure to indirect microbiota and their metabolites from urine and prostatic tissues (6). Over the past decade, there has been a surge in interest in the potential role of the microbiome to both promote and inhibit tumor growth as well as its therapeutic potential. Such interest has been further fueled by the promising discovery of distinct microbial signatures in cancer patients and the mounting evidence supporting the use of these signatures as biomarkers for diagnosis and risk stratification.

The advancement of sequencing technology has enabled studies of microbiota in prostate tissues. Several studies have reported a correlation between microbiota and the incidence and metastasis of PC (7–10). Interactions between microbiota and the prostate occur through direct or indirect mechanisms (11–13). While previous research has primarily focused on the effects of the gut microbiome on PC through immune modulation and metabolic processes, there is currently insufficient evidence to establish a causal relationship between gut microbiome and PC. Moreover, it remains unclear whether identified microbes are genuinely linked to cancer or simply coincidental. The microbiota present in the prostate tissue and urinary tract can directly influence PC through chronic inflammatory conditions such as chronic prostatitis and benign prostatic hypertrophy (BPH). Although the direct influence of microbiota in tissue or urine samples can provide more insight into the interaction in PC, identifying microbiota in these samples is challenging due to potential contamination that can cause bias in low-biomass samples studies (14, 15).

Previous attempts to define the microbiota in prostate tumor tissues have been inconclusive. A study of a Chinese cohort of 65 radical prostatectomy (RP) specimens found no difference in microbiota between tumor and benign tissues, showing that genera *Escherichia*, *Cutibacterium*, *Acinetobacter*, and *Pseudomonas* were commonly abundant in all tissues (8). These genera are known to be common laboratory contaminants (15), which could explain the lack of difference in microbiota between tissue types. A recent study analyzed 94 patients with PC who underwent RP and found that *Bacteroides fragilis*, *Staphylococcus saprophyticus*, and *Vibrio parahaemolyticus* were less abundant whereas *Shewanella* was more abundant in malignant tissues (10). However, the study did not control for potential contaminants. It is worth noting that host factors can influence microbiota studies related to human diseases (16). Unfortunately, few studies have considered host variables when analyzing tissue microbiota in PC. Therefore, it is necessary to study the microbiota in PC tumor tissues while controlling for contaminants and considering host variables.

Ethnicity may influence the microbiome signature, which could reflect differences in cancer characteristics. Recent studies have reported that Asian patients with PC exhibit higher Gleason scores, more advanced stages at diagnosis, and poorer prognostic indicators compared to other ethnic groups. A comparative analysis of PC outcomes across different ethnic groups revealed that Asians exhibited a significantly higher likelihood of presenting with aggressive disease phenotypes, characterized by a higher rate of extraprostatic extension and seminal vesicle invasion (17). Furthermore, Asians possess a distinctive molecular profile that may contribute to the aggressive nature of the disease, including variations in androgen receptor signaling pathways and differential expression of oncogenic drivers (18). These findings underscore the necessity of microbiome analysis in prostate tissues from Korean patients with PC.

Roles of local microbial pathways in the development and progression of PC remain unclear. Therefore, the objective of this study was to analyze microbiota in malignant tumors and adjacent benign tissues after removing potential contaminants during the process and considering host variables to identify differences in microbiota and their potential roles in PC.

## 2 Materials and methods

### 2.1 Ethics statement

This retrospective and multi-institutional study was approved by the Institutional Review Board (IRB) of Seoul National University Bundang Hospital (Approval number: B-1910-570-302) in accordance with ethical standards of the 1964 Declaration of Helsinki and its later amendments. All patients enrolled in this study provided informed consent. Personal identifiers were completely removed and data were analyzed anonymously.

### 2.2 Study subjects and sample collection

Biopsy-proven and treatment-naïve PC and matched benign tissues were collected from robot‐assisted laparoscopic radical prostatectomy (RARP) performed by a single surgeon (S.K.H.). Inclusion criteria were: (1) solitary and the Prostate Imaging Reporting and Data System (PI-RADS) score 4 or 5 (visible) on multi-parametric MRI (mpMRI), (2) unilateral disease on prostate biopsy, (3) good correlation with whole-mount sections, and (4) lymph node dissection for significant lesion on mpMRI. We simultaneously collected benign and malignant tissues in the operation room during RALP. Most PC lesions were distinguishable from normal tissues under direct vision. Benign tissue samples were obtained from areas separate from PC tissues. Lymph node samples were also obtained in case of a significant lesion on mpMRI and/or suspicion of lymph node metastasis during surgery. Each tissue sample was obtained in the form of cube-shaped pieces, with dimensions of at least 5 mm. All procedures were performed under strict aseptic conditions using sterilized surgical tools and a tube holder at every single step. To ascertain the absence of microbial contamination in the operation room, an empty cryotube filled with phosphate-buffered saline was utilized. The cap of the tube was left open in proximity to the sampling tube throughout the surgical procedure. It was closed at the end of the surgery. Negative controls (for check contamination) were also analyzed along with tissue samples. Collected samples were immediately frozen at −80°C, without any additives, until further processing.

### 2.3 Clinicopathologic parameters

Clinicopathological data including patient demographics, age at surgery, body mass index (BMI), past medical history including type II diabetes mellitus (DM) and hypertension (HTN), clinical stages, preoperative biopsy profiles, laboratory tests including prostate-specific antigen (PSA), radiologic findings of mpMRI and/or transrectal ultrasound (TRUS), pathologic stages, and Gleason score (GS) were prospectively collected. Risk groups were defined according to the National Comprehensive Cancer Network (NCCN) guidelines (19): low-risk group, T1-T2a, GS ≤ 6, and PSA < 10 ng/mL; intermediate-risk group, T2b-T2c or GS = 7 or PSA = 10-20 ng/mL; and high-risk group, T3a or GS = 8-10 or PSA > 20 ng/mL. Histological evaluations were conducted in accordance with the International Society of Urological Pathology (ISUP) protocol by a pathologist with expertise in genitourinary pathology.

### 2.4 DNA extraction and 16S rRNA gene amplicon sequencing

Total DNAs were extracted from benign and malignant tissues (cube-shaped whole pieces of tissue) using the RNeasy PowerMicrobiome Kit (Qiagen, Valencia, CA, USA) and quantified using a BioPhotometer D30 with μCuvette G1.0 (Eppendorf, Hamburg, Germany). Bacterial 16S rRNA gene (targeted V1-V3 hypervariable regions) was amplified using a C1000 thermal cycler (Bio-Rad, Hercules, CA, USA) based on the protocol for preparing a 16S metagenomic sequencing library for a MiSeq system (Illumina, San Diego, CA, USA) as previously described (20, 21). The amplification was performed under the following conditions: initial denaturation at 95°C for 3 min; 25 cycles of denaturation at 95°C for 30 s, annealing at 55°C for 30 s, and extension at 72°C for 30 s; and a final extension at 72°C for 5 min. Purification and size selection were conducted using HiAccuBead (AccuGene USA, San Diego, CA, USA). Briefly, vortexed HiAccuBeads (0.8 × volume for 600-700 bp target size) were added to each PCR product tube, mixed, and incubated at room temperature for 5 min. The tubes were then placed on a magnetic stand for 2 min, and the supernatant was carefully removed and discarded. Each sample was washed twice with 80% ethanol, and 52.5 µl of 10 mM Tris (pH 8.5) was added to each tube. The mixture was incubated at room temperature for 2 min, followed by a 2 min incubation on the magnetic stand. Finally, 50 µl of the supernatant was transferred to a new tube. Index PCR reactions were performed using 5 µl of purified PCR product in a final volume of 50 µl with the Nextera XT index kit (Illumina). Amplicons from each sample were purified again using HiAccuBead (AccuGene). The concentration of each library was measured using the Takara PCR Thermal Cycler Dice Real Time System III (Takara Bio, Otsu, Japan) with the GenNext NGS Library Quantification Kit (Toyobo, Osaka, Japan). Equimolar concentrations (10nM) of each library were pooled and sequenced on an Illumina MiSeq system (300-bp paired ends) in accordance with the manufacturer’s instructions. Negative controls were included at every step to check contamination. A total of 64 negative controls were sequenced with samples. These sequenced negative controls included 58 empty cryotubes for the purpose of monitoring the operation room (sampling blank), three DNA-free water samples added to the RNeasy PowerMicrobiome Kit (negative extraction control), and three DNA-free water samples added to the amplicon library preparation kit (library negative controls). For the sampling blank, DNA was extracted after washing the inside of the blank sampling tube with 250 µl of buffered saline. For the negative extraction control, 250 µl of DNA-free water added to the extraction kit. The preparation of library for negative controls was conducted in accordance with above process, and the average pooled volume of tissue sample libraries was added for sequencing.

### 2.5 Amplicon sequence analysis

Raw reads of amplicon sequences were analyzed using the QIIME2 pipeline (ver. 2020.11.01) (22). Briefly, raw sequences were quality filtered and denoised using DADA2 as implemented in QIIME2. The output file of DADA2 was a feature table including all amplicon sequence variants (i.e., ASVs table). Samples with fewer than 10,000 sequence reads were excluded from further analysis. Taxonomic identification of representative sequences was conducted using the BLAST classifier with the EzTaxon-e database (23). The Decontam package in R software (ver. 4.0.3) was employed to reduce potential contaminants of low biomass samples based on sequence data in negative controls (24). The identification and removal of potential contaminants were achieved using prevalence-based algorithms with a threshold set at 0.1. Diversity indices were calculated using the DADA2 feature table after rarefaction without replacement.

### 2.6 Whole metagenome sequencing and analysis

Whole metagenome sequencing was conducted for normal and tumor tissues from 21 patients with DM. Extracted total DNAs were fragmented using NEBNext dsDNA Fragmentase (NEB) according to the manufacturer’s protocol (fragment size: 300-500 bp). A total of 30 ng of fragmented DNA was utilized as the input for each library preparation. Metagenomic libraries were prepared using the ThruPLEX DNA Sequencing Kit (TaKaRa Bio USA, Inc., CA, USA) according to the manufacturer’s protocol. Sizes of metagenomic libraries were verified using Bioanalyzer 2100 (Agilent Technologies). Equimolar concentrations of each library from different samples were pooled and sequenced using an Illumina NovaSeq system (250 bp-paired ends).

For whole metagenome analysis, adapter removal and quality filtering were conducted using Trimmomatic v.0.39 with default options (leading and trailing: 3; sliding window size: 4; quality: 15; min length: 150). These paired-end sequences were merged using PEAR v.0.9.11 (25). The presence of human genes was identified and removed using BBmap (http://sourceforge.net/project/bbmap) with a reference human genome. Taxonomic profiles for each sample were determined using Kraken2 v.2.1.2 (26). Filtered reads were normalized for total marker-gene length and outliers, marker-gene presence/absence, and marker gene abundance (in reads per kilobase per million units; RPKM). Functional metagenome profiling was conducted using HUMAnN3 v. 3.6 (27). Metabolic pathways were analyzed based on the MetaCyc database. A total of 928,910,793 reads (median 14,982,432 reads per sample) were obtained from whole metagenome sequencing.

### 2.7 Statistical analysis

Chi-square test was used to assess the significance of differences in categorical variables between groups. Statistical significance of differences in taxonomic and functional features between groups was determined using the Mann-Whitney U test in the R software (ver. 4.1.3). Microbiota differences were visualized using non-metric multidimensional scaling (NMDS) plots based on Bray-Curtis distance. Effect size and significance of each covariate in the variation of microbiota were calculated using the *‘envfit’* function in the R package vegan (v.2.5-7). Significantly different taxonomic and functional features were visualized using a heatmap and a boxplot. Significantly different functional pathways between intermediate and high pathologic groups were visualized using the *‘ggreple’* R package. Network analysis was conducted to identify correlations between functional pathways and genera. Node size was scaled according to the centrality measure, which was determined using the ‘*igraph*’ package. Edges were determined using Spearman’s rank correlation coefficient to depict correlations between genera and functional pathways. The corresponding correlation network was visualized using the ‘*ggraph*’ package. Normalized abundances of significantly different pathways between intermediate and high pathologic groups in malignant tissues were compared to those in benign tissues using the Sankey diagram plot with the *‘networkD3’* R package. Results with *p* < 0.05 were considered statistically significant.

## 3 Results

### 3.1 Comparison of microbiota between different tissues from patients with prostate cancer reveals minor differences depending on the tissue type and pathological stage

A total of 63 patients were enrolled in this study. Tissue samples obtained from three patients were excluded due to temperature criteria during the transfer process. Clinicopathological characteristics of 60 subjects for microbiota analysis are summarized in **Table 1**. The mean age of subjects was 66.8 ± 7.7 years old. Their mean BMI was 25.0 ± 3.3. Additionally, 35.0% of patients had a DM and 73.3% had multicentricity.

**Table 1:** Clinicopathological characteristics of study subjects.

Characteristic	
Total number	60
Age (years)	66.8 ± 7.7
Height (cm)	167.3 ± 5.4
Weight (kg)	69.9 ± 9.0
BMI (kg/m^2^)	25.0 ± 3.3
Diabetes Mellitus (DM)	
No (n, %)	39 (65%)
Yes (n, %)	21 (35%)
Prostate-specific antigen (PSA)	
low (< 10 ng/mL) (n, %)	32 (53.3%)
intermediate (10 - 20 ng/mL) (n, %)	11 (18.3%)
high (> 20 ng/mL) (n, %)	17 (28.3%)
Gleason score (GS)	
low (< 7) (n, %)	0 (0%)
intermediate (= 7) (n, %)	41 (68.3%)
high (> 7) (n, %)	19 (31.7%)
Pathologic stage	
low (T1-T2a) (n, %)	2 (3.3%)
intermediate (T2b-T2c) (n, %)	24 (40%)
high (> T3a) (n, %)	34 (56.7%)
Multicentricity	
No (n, %)	16 (26.7%)
Yes (n, %)	44 (73.3%)

We analyzed 118 tissue samples (59 benign and 59 malignant tissues) from 60 patients, along with 64 negative control samples (58 sampling, 3 DNA extraction, and 3 library preparation controls). Two samples (one benign tissue and one malignant tissue) were excluded from the 120 collected tissue samples due to low quality (median Q < 30) and insufficient read number (< 10,000) of obtained sequences. Following quality control procedures, a total of 23,804,680 sequence reads (a total of 8,817,472 reads with an average of 149,448.7 reads per sample for benign tissue samples; a total of 7,688,979 reads with an average of 130,321.7 reads per sample for malignant tissue samples; and a total of 7,298,229 reads with an average of 114,034.8 reads per sample for negative controls) were subjected to analysis. To ensure the quality of sequences and to remove potential contaminants, the Decontam package was used to trim sequences identified in negative controls. The diversity of microbiota detected in negative controls was found to be significantly lower than that in tissue samples (*p* < 0.001; **Supplementary Figure S1**). Intra-variations of tissue samples were found to be significantly lower than intra-variations of negative control samples and inter-variations between tissue samples and negative controls (*p* < 0.001). Nine genera were removed from microbiota in tissue samples using the Decontam process (**Supplementary Figure S1C**, **Supplementary Table S1**).

**Figure 1:**
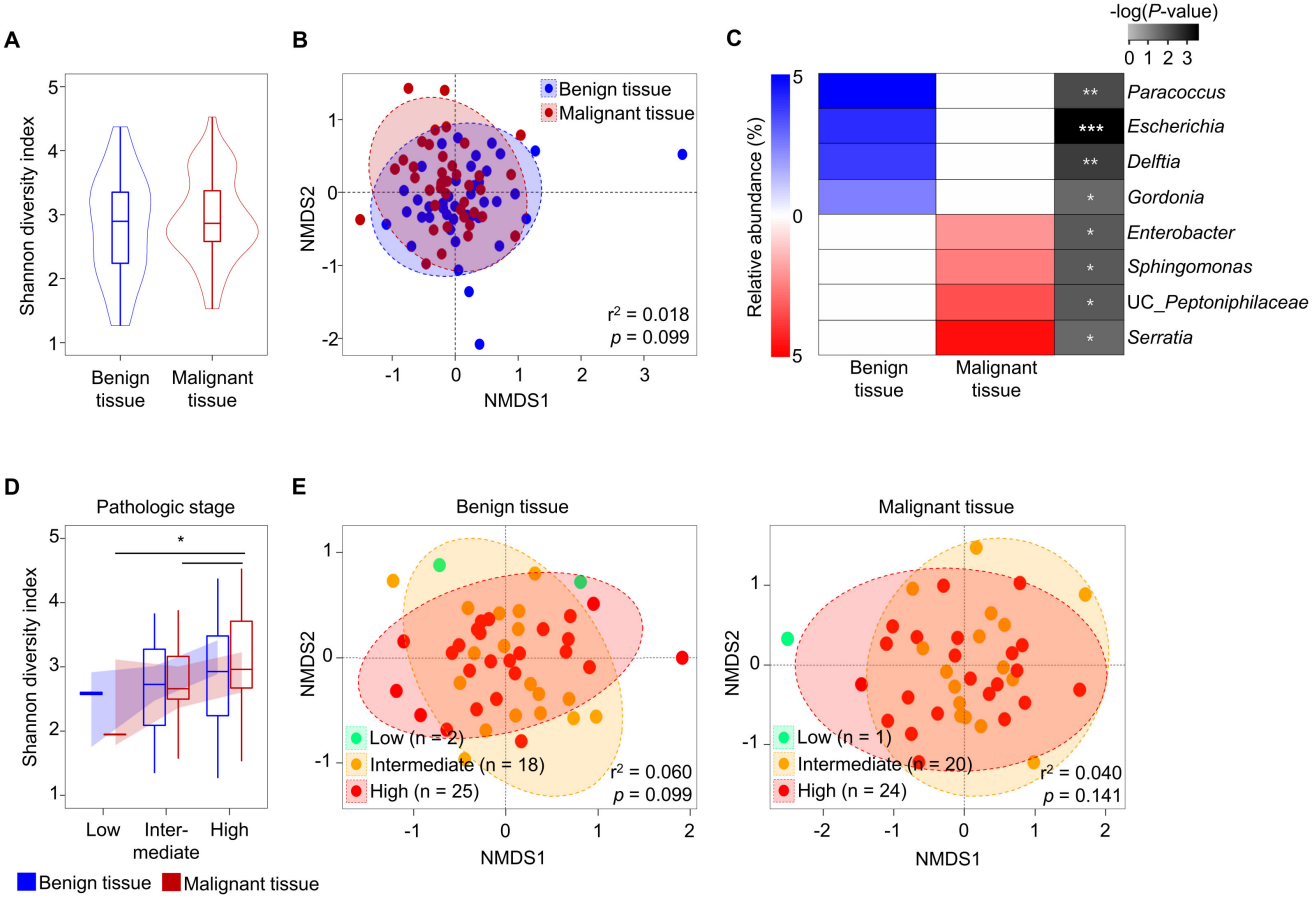
Comparison of tissue microbiota in patients with prostate cancer. **(A)** Shannon diversity index of microbiota was compared between benign and malignant tissues. **(B)** Compositions of microbiota were compared between benign and malignant tissues based on the Bray-Curtis distance. **(C)** Relative abundance of different genera in the microbiota between benign and malignant tissues. The significance was calculated by Mann-Whitney test. **(D)** Shannon diversity index of microbiota was compared according to pathologic stage. **(E)** Compositions of microbiota in benign and malignant tissues were compared among pathologic stage groups based on the Bray-Curtis distance. *p < 0.05, **p < 0.01, ***p < 0.001.

Covariates significantly associated with variations of tissue microbiota were analyzed using the EnvFit model after trimming potential contaminants (**Supplementary Table S2**). The variation of microbiota was significantly associated with tissue type (r^2^ = 0.036, *p* < 0.05). However, there were no significant differences in Shannon diversity or beta-diversity of microbiota between benign and malignant tissue samples (**Figures 1A, B**; *p* > 0.05). Although there was no significant difference in diversity between tissue types, relative abundances of eight genera differed significantly (**Figure 1C**; *p* < 0.05). *Paracoccus*, *Escherichia*, *Delftia*, and *Gordonia* were more abundant in benign tissues than in malignant tissues, while *Serratia*, uncultured (UC)_*Peptoniphilaceae*, *Sphingomonas*, and *Enterobacter* were more abundant in malignant tissues than in benign tissues.

The aggressiveness of prostate cancer may influence tissue microbiota (28). In this study, we analyzed tissue microbiota based on aggressiveness indices (such as PSA, GS, and pathologic state) and multicentricity. Our findings indicated that the diversity of tissue microbiota increased with the severity of cancer in both benign and malignant tissues. However, these changes were only significant in the pathological stage (*p* < 0.05; **Figure 1D**, **Supplementary Figure S2**). There were no significant differences in beta-diversity of microbiota according to pathological stage (*p* > 0.05; **Figure 1E**). Furthermore, there were no significant differences in aggressiveness status between benign and malignant tissues (**Supplementary Figure S3**).

### 3.2 The status of DM significantly influences microbiota in malignant tissues

**Figure 2:**
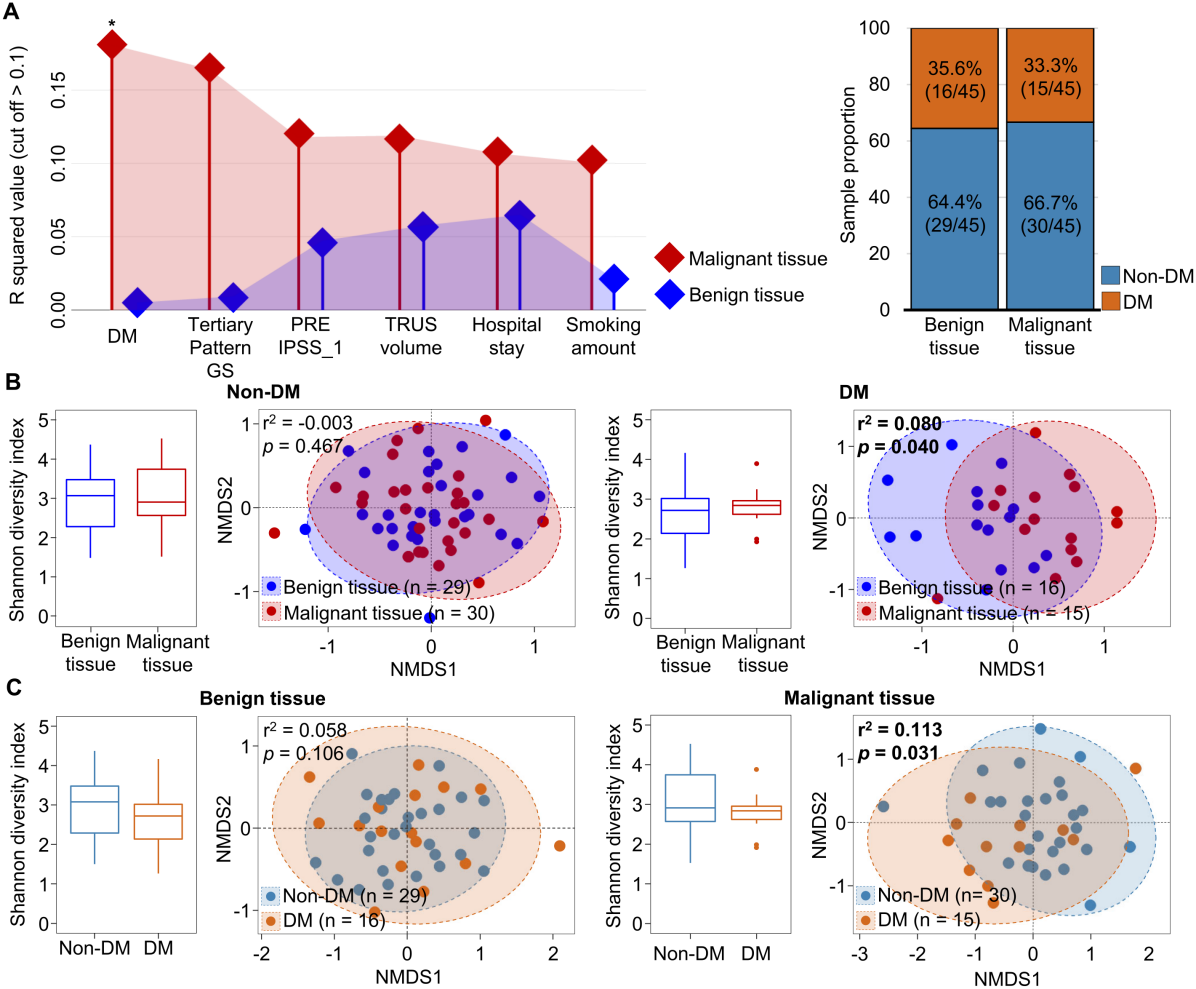
Comparison of tissue microbiota in patients with prostate cancer according to diabetes status. **(A)** Covariate factors that correlated with variations of tissue microbiota were determined using the EnvFit model. Analyzed numbers of patients with diabetes were compared between benign and malignant tissues. **(B)** Diversity and compositions of microbiota were compared between benign and malignant tissues in non-DM and DM groups. **(C)** Diversity and compositions of microbiota were compared between non-DM and DM group of benign and malignant tissues. The significance in NMDS plots was calculated by ANOSIM based on the Bray-Curtis distance. DM, diabetes mellitus; GS, Gleason score; IPSS, international prostate symptom score; TRUS, transrectal ultrasonography; NMDS, non-metric multidimensional scaling; ANOSIM, analysis of similarities. *p < 0.05.

Although microbiota variation in tissue samples of prostate cancer was influenced by tissue type, no clear distinction was observed in alpha- and beta-diversity comparisons between tissue types, even when considering aggressiveness. Therefore, we analyzed factors influencing the variation of microbiota in each tissue type using the EnvFit model (**Figure 2A**). The clinical features of DM, tertiary pattern GS, preoperative international prostate symptom score (IPSS), TRUS volume, hospital stay period, and smoking amounts were identified as having a greater impact on the variation of tissue microbiota in malignant tissues than in benign tissues. The status of DM was found to significantly affect microbiota in malignant tissues (*p* < 0.05). Despite similar numbers of samples with DM in both benign and malignant tissues, microbiota in benign tissues were not affected by the DM status. These results indicate that DM status only affects microbiota in malignant tissues. Therefore, we analyzed alterations of tissue microbiota based on DM status in both malignant and benign tissues (**Figure 2B**).

In patients with DM and those without DM, the alpha diversity of microbiota was not significantly different between benign and malignant tissues. However, the beta diversity of microbiota was significantly different between benign and malignant tissues in DM patients (r^2^ = 0.082, *p* < 0.05). Analysis of the influence of DM status on the microbiota in each tissue also showed that the microbiota in malignant tissue was significantly different between patients with DM and those without DM (r^2^ = 0.113, *p* < 0.05; **Figure 2C**). These results indicate that the microbiota in malignant tissue is influenced by DM status. Clinicopathological factors were compared between non-DM and DM groups (**Supplementary Table S3**). High GS score and multicentricity were found to be significantly different between the two groups (*p* < 0.05). Other factors did not differ significantly between the two groups.

**Figure 3:**
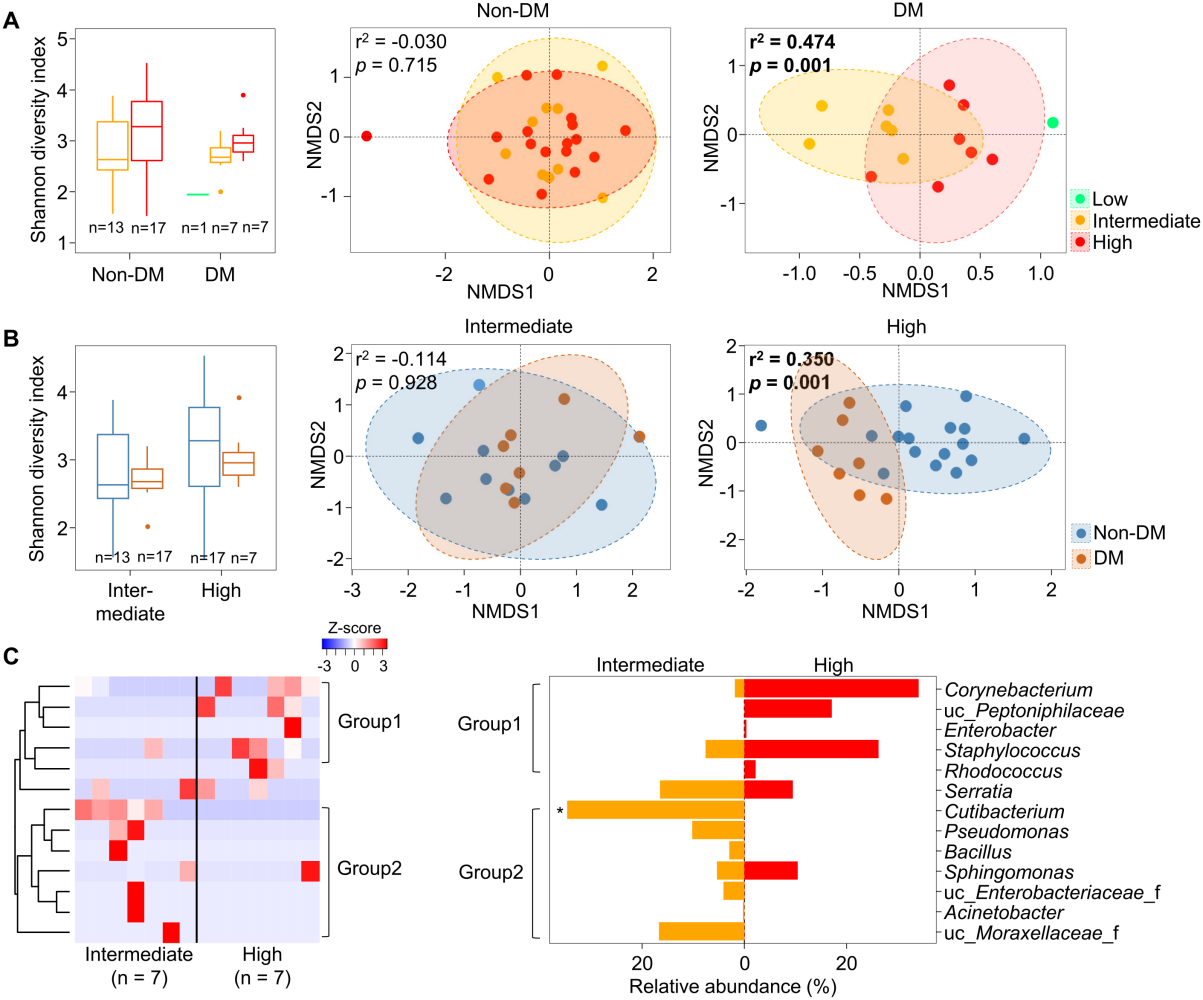
Microbiota in malignant tissues were compared according to pathologic stage. **(A)** Diversity and compositions of microbiota were compared among pathologic stages in non-DM and DM groups. **(B)** Diversity and compositions of microbiota were compared between non-DM and DM groups in intermediate and high pathologic groups. The significance in NMDS plots was calculated by ANOSIM based on the Bray-Curtis distance. **(C)** Differences of genera in microbiota between intermediate and high pathologic groups. Genera within group 1 were higher in the high pathologic groups while genera within group 2 were higher in the intermediate group. The clustering was conducted using Spearman’s rank correlation coefficient. DM, diabetes mellitus; NMDS, non-metric multidimensional scaling; ANOSIM, analysis of similarities. *p < 0.05.

Since microbiota in malignant tissues were influenced by DM status, we analyzed alterations of microbiota in malignant tissues by the aggressiveness of prostate cancer according to DM status. Our findings revealed that the pathologic stage significantly affected the alteration of microbiota in malignant tissues from patients with DM (r^2^ = 0.474, *p* = 0.001), but not in patients without DM (**Figure 3A**). The influence of DM on the difference in microbiota according to pathologic stage was significant in the high pathologic stage group (r^2^ = 0.350, *p* = 0.001; **Figure 3B**). Different genera between intermediate and high pathologic stage groups were divided into two groups (**Figure 3C**). Genera within group 1, including *Corynebacterium* and *Staphylococcus*, were more abundant in the high pathologic group than in the intermediate group. In contrast, genera within group 2, including *Cutibacterium* and *Pseudomonas*, were more abundant in the intermediate group than in the high pathologic group. The difference in *Cutibacterium* was significant between the two groups (*p* < 0.05). However, other aggressiveness statuses did not influence the alteration of microbiota in malignant tissues according to DM status (**Supplementary Figure S4**). These findings indicate that microbiota in malignant tissues with a high pathological stage exhibit significantly different compositions according to DM status.

### 3.3 Whole metagenome analysis reveals potential influences of microbiome in malignant tissues from patients with DM

**Figure 4:**
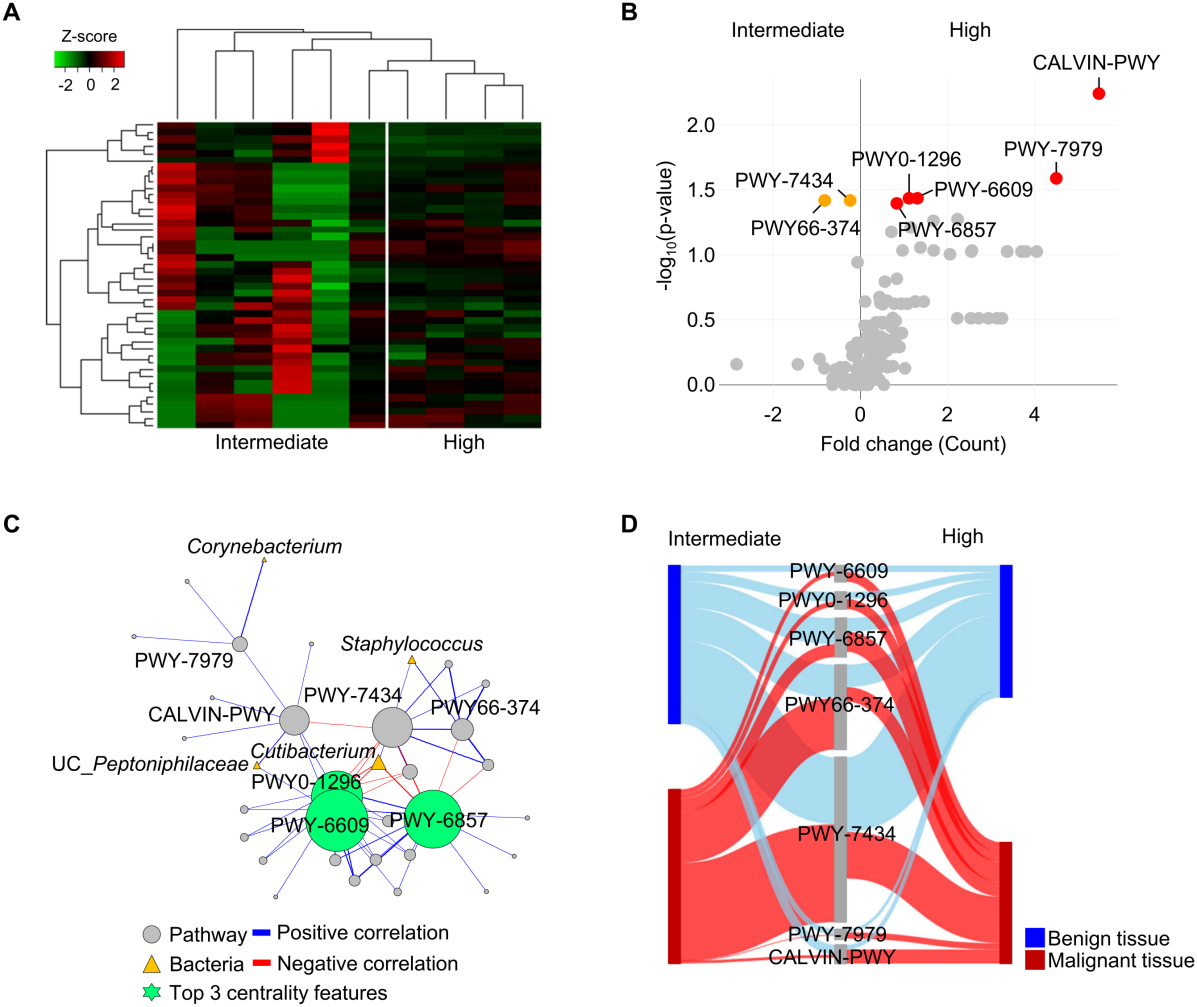
Functional features of tissue microbiome were compared between intermediate and high pathologic groups in malignant tissues of patients with diabetes. **(A)** Profiles of functional features in microbiome were compared between intermediate and high pathologic groups in the heatmap analysis. The clustering was conducted using Spearman’s rank correlation coefficient. **(B)** A volcano plot showing different functional features of tissue microbiome between intermediate and high pathologic groups. Functional features with *p* < 0.05 were considered significant features. **(C)** Potential interactions between pathways and bacteria were analyzed by network analysis. Positive and negative correlations are marked by blue and red edges, respectively. Node sizes were scaled on the eigenvector centrality measure. Only significant correlations with *p* < 0.05 are shown. Pathways are marked by grey circle and bacteria are marked by yellow triangle. Top 3 centrality features are marked by green circle symbol. Pathways are identified based on the MetaCyc database. **(D)** Relative abundances of significantly different pathways were compared between benign and malignant tissues using the Sankey diagram plot.

The present study revealed alternation of microbiota in malignant tissues of patients with DM and influence of pathological stage on microbiota. To identify the potential role of the microbiome in malignant tissues according to pathological stage, whole metagenome sequences of 42 tissues (21 benign and 21 malignant tissues) from 21 patients with DM were analyzed. Profiles of microbiome pathways in malignant tissues were clearly separated between intermediate and high pathologic groups (**Figure 4A**). Seven pathways were found to be significantly different between the two groups (*p* < 0.05; **Figure 4B**). Normalized abundances of five pathways, PWY-6857 (retinol biosynthesis), PWY0-1296 (purine ribonucleosides degradation), PWY-6609 (adenine and adenosine salvage III), PWY-7979 (protein O-mannosylation III), and CALVIN-PWY (calvin-benson-bassham cycle), were found to be significantly increased in the high pathologic group compared to those in the intermediate group, whereas two pathways, PWY-7434 (terminal O-glycans residues modification) and PWY66-374 (C20 prostanoid biosynthesis), were found to be increased in the intermediate group compared to those in the high pathologic group.

Interactions between pathways and bacteria in malignant tissues were analyzed using network analysis (**Figure 4C**). Correlations with significance (*p* < 0.05) were lined and the importance of detected features was determined by centrality scores. Increased pathways (PWY-6609, PWY0-1296, and PWY-6857) in the high pathologic groups were identified as keystone features in the interaction network. These pathways were negatively correlated with *Cutibacterium*, which was reduced in the high pathologic group compared to that in the intermediate group. Relative abundances of significantly different pathways were compared between benign and malignant tissues (**Figure 4D**). Abundances of pathways were similar between groups in benign tissues, whereas their significantly altered abundances were detected in malignant tissues. These results indicate that the altered microbiome in malignant tissue could influence both tissue environment and host tissue.

## 4 Discussion

In this study, tissue microbiota and their functional genes in patients with PC were analyzed and compared between benign and malignant tissues after removing potential contaminations. Results indicated that the microbiota present in malignant tissue was significantly influenced by the DM status of patients. In patients with DM, microbiota were clearly distinguished between benign and malignant tissues, with the influence of pathologic stage being significant in malignant tissues. Moreover, the potential role of the microbiome significantly differed according to pathological stages of patients with DM. These results indicate that tissue microbiome is influenced by diabetes in patients with PC and that an altered microbiome may affect the microenvironment of prostate tissues.

Recent studies have reported the presence of unique microbiota and their association with cancer prognosis regarding several cancer types (29, 30). Different signatures of microbiota between benign and malignant prostate tissues have been identified in previous studies (10, 31). The microenvironment of prostate tissue can influence the microbiota of tissue, which in turn can affect tumor growth or progression through different mechanisms such as immune modulation and extracellular matrix remodeling. Enterobacteriaceae can modify the extracellular matrix by secreting enzymes such as alkaline proteases and elastases (32). Uro-pathogenic strains of *E. coli* have been shown to induce prostate tissue damage in rat models of prostatitis (33), which is mediated by cytotoxic necrotizing factor 1 (CNF1), a virulence factor that can promote PC progression (34). *Listeria monocytogenes*, *Methylobacterium radiotolerans* and *Xanthomonas albilineans* were negatively correlated with Gleason score, Tumor-Node-Metastasis (TMN) stage, and PSA level, respectively (28). *L. monocytogenes* has been reported to play anti-tumor roles in PC by stimulating innate and adaptive immune response (35). Nevertheless, distinct bacteria were identified in each study, showing no significant differences between tumor tissue types in several studies (8, 36). Our findings in the comparison of microbiota between benign and malignant tissues differed from those of previous studies. This discrepancy might be attributed to a number of factors, including ethnicity, age, disease severity (37), detection methods, and potential contaminants in sequencing-based studies for low-biomass samples such as tissues (6). To prevent biased results due to potential contamination during sequencing-based analysis, we used 64 negative controls to eliminate any possible contaminant sequences. The Decontam pipeline identified nine genera as contaminants by comparing detected sequences between negative controls and tissue samples (**Supplementary Table S1**). Removing potential contaminants from sequencing results is a crucial step in each study, as contaminants can vary between studies due to differences in reagents and experimental procedures. Furthermore, host covariates have been reported to be important in disease-related microbiome analysis (16). However, previous studies have not explicitly considered covariates of patients, including DM status. Nevertheless, previous studies have indicated the existence of a non-sterile prostate microenvironment and suggested that dysbiosis of the microbiome may play a role in cancer progression.

Our results showed a minor difference of microbiota in prostate tissues even after removing potential contaminants. However, the significant correlation between tissue microbiota and PC can be identified and characterized when considering DM status as a host covariate. Microbiota in benign and malignant tissues exhibited a significant difference in patients with DM. Furthermore, the influence of DM on microbiota in prostate tissues was significant in malignant tissue and in the high pathological group. Although an inverse association between DM and the development of PC has been reported, the mortality rate from PC is higher in patients with DM than in those without DM (38–40). The risk of PC death is increased among men using antidiabetic drugs compared with nonusers (41). These findings suggest that DM can affect the progression rather than the initiation of PC. Elevated androgen receptor signaling and activity due to altered insulin/IGF-1 receptors could be related to the high mortality rate in PC patients with DM (42). In addition, hyperglycemia could decrease docetaxel-induced apoptosis, resulting in a poor response to chemotherapy (43). The association between DM and various cancers, including bladder cancer, has been reported (44). However, the underlying mechanisms connecting DM and PC remain to be established. Microbiota in tissues might be influenced by DM as a consequence of changes in cancer cell metabolism to anaerobic glycolysis, known as the Warburg effect (45).

The initial study suggesting a strong association between human gut microbiome and type 2 DM was reported in 2010 (46). Since then, numerous studies have reported an association between DM and the distribution of the gut microbiome via metabolite, immunologic, and neuroendocrine pathways (47, 48). Furthermore, dysbiosis of microbiota resulting from DM has been proposed as a potential risk factor for therapeutic resistance in other malignancies (49). To the best of our knowledge, studies investigating the impact of DM on microbiota within prostatic tissues have not been reported yet. Our results indicated that there were minor differences in simple comparison of microbiota between benign and malignant tissue in PC patients. However, we found that the presence of DM in PC patients not only significantly changed tissue microbiota, but also affected interactions between microbiota and prostate tissues, which might result in disease progression or a pathological status. These findings indicate that a novel biomarker for DM-induced prostate cancer tissue and novel therapeutic avenues can be developed by utilizing microbiome data.

Previously, it has been reported that changes in specific microbiota are associated with the progression of prostate cancer (10). In addition, *Cutibacterium* has frequently been detected in prostate tissues in both culture-based and culture-independent studies (8, 31, 50). *Cutibacterium* is a commensal bacterium in the skin. It can be associated with chronic inflammation in the prostate of patients with PC (50). Acute and chronic inflammation can be induced by human prostatectomy derived *Cutibacterium acne* isolates through pro-inflammatory pathways in murine models (51). *C. acne* can induce cell proliferation and secretion of cytokines and chemokines such as IL-6 and IL-8 in *in vitro* assays (52, 53). These studies provide a possible contribution of *Cutibacterium* in the development or progression of PC. In our study, the relative abundance of *Cutibacterium* was significantly higher in the intermediate pathologic group than in the high pathologic group. This result suggests that *Cutibacterium* might be related to inflammation, particularly in the intermediate pathologic group, while other bacteria might be related to aggressiveness, particularly in the high pathologic group.

The analysis of functional features obtained from whole metagenome sequences revealed differences in microbiome interactions with prostate tissues between intermediate and high pathologic groups. Seven pathways were found to be significantly different between intermediate and high pathologic groups. Terminal O-glycans residues modification pathway (PWY-7434), which exhibited a higher microbial gene count in the intermediate group than in the high pathologic group, was positively correlated with *Cutibacterium*. A previous study has indicated that alterations in O-glycans in prostate cancer are associated with cancer progression, including the induction of androgens in prostate cancer tissues and elevated serum levels of PSA (54). Additionally, O-glycans have been demonstrated to be up-regulated in early prostate cancer tissues but down-regulated following cancer progression (55). These results might be related to the higher abundance of *Cutibacterium* in the intermediate group than in the high pathologic group in our study.

*Corynebacterium*, *Staphylococcus*, and UC_Peptoniphilaceae were positively correlated with significantly abundant pathway genes in the high pathologic group. Three pathways (PWY-6609, PWY0-1296, and PWY-6857), which were significantly abundant in the high pathologic group, were important factors affecting interactions between bacteria and functional pathways in malignant tissues. Pathways of adenine, adenosine salvage (PWY-6609) and purine ribonucleosides degradation (PWY0-1296) might be related to DNA damage resulting from PC progression. Retinoid is a synthetic and biological molecule similar to vitamin A. Effects of retinoid and their derivatives on tumor tissues are controversial. Although potential anticarcinogenic effects of retinoids, including induction of apoptosis, cell differentiation, inhibition of proliferation, and enhancement of immune surveillance, have been reported (56), there is also evidence that retinoids can enhance tumor growth (57, 58). Our results also indicated that retinol biosynthesis (PWY-6857) was significantly abundant in the high pathologic group as an important pathway in malignant tissues. Although differences in these pathways between intermediate and high pathologic groups were not observed in benign tissues, they were detected in malignant tissues of patients with DM. Since glucose serves as a primary energy source for the growth and development of prostate cancer cells in the body, advancement of prostate cancer might have been hastened in individuals with DM. These results indicate that the microbiome in prostate tissues might influence the host in malignant tissues according to aggressiveness, particularly in patients with DM.

Results of this study are crucial for future research into prostate cancer and tissue microbiome, offering new insights into interactions between metabolic diseases such as DM and prostate cancer microenvironment. While previous studies have demonstrated that DM can accelerate prostate cancer progression, specific changes based on pathological stage have not been identified. Notable differences in microbial presence and activity between intermediate and high-grade prostate cancer in DM patients suggest a connection between certain microbial patterns and the severity of cancer. The topic of a direct or indirect impact of microbiota on the initiation and prognosis of a variety of malignancies, including PC, has recently attracted an intense interest (30). It has been recognized that microbiota can influence the proliferation of tumors and responses to therapies directly or indirectly by involving immune modulation, metabolic changes, and epithelial damage (59). Therefore, our findings can lead to targeted management approaches for PC in DM patients, potentially enhancing outcomes by tackling both microbial and metabolic factors. Future studies should investigate influence of the microbiome on cancer progression and consider microbiome-based treatments as an innovative aspect of prostate cancer therapy strategies.

This study has several limitations. First, we used adjacent benign tissue samples to compare with microbiota in malignant tumor tissues. Obtaining normal prostate tissue from healthy subjects is challenging due to ethical considerations. Second, the number of subjects included in this study was relatively small and the comparison of microbiome was conducted as a cross-sectional study. Further studies with a larger sample size and longitudinal approaches are needed to validate findings in this study. However, our analyzed sample size was comparable to previous studies and a repeated collection of prostate tissue samples was not feasible. Notwithstanding these limitations, our study provides insights into the influence of DM on the microbiome in prostate tissues of patients with PC and the potential influence of tissue microbiome on the host according to pathological severity. In addition, our results reduced the potential contaminants using sequences in negative controls and the Decontam program to improve previously indicated contaminants in tissue sample analysis. Our findings have the potential to inform the development of novel treatment or prediction of PC with consideration of microbiome information.

To the best of our knowledge, this study is the first to demonstrate the influence of DM on the microbiota in prostate tissues of patients with PC. The microbiota and their potential functions significantly differ between benign and malignant tissues according to pathologic severity in patients with DM. Our findings indicate that the altered microbiome in prostate tissues may affect the microenvironment in tissues and the progression of PC. These results extend the evidence for the acceleration of PC progression by DM in prostate tissues and the influence of the microbiome on the progression of PC. This study provides also valuable insight for microbiome research in PC and clinical approaches to improve therapy direction and prediction of progression.

## Data Availability

The sequence reads obtained from this study are available in the EMBL SRA database under the accession number PRJEB74558 (http://ebi.ac.uk/ena/data/view/PRJEB74558).
