# Supplementary material for: Influence of diabetes on microbiome in prostate tissues of patients with prostate cancer
Source: Front Oncol. 2024 Aug 16;14:1445375. doi: 10.3389/fonc.2024.1445375 (PMC11365045; doi:10.3389/fonc.2024.1445375)
Supplement: Supplementary file 1 [file DataSheet1.docx]

Supplementary Material

**Supplementary Table S1.** The list of amplicon sequence variants (ASVs) from sequence data of samples determined by the Decontam pipeline (prevalence threshold = 0.1)

| Phylum | Class | Order | Family | Genus | *p*-value | Contaminant |
| --- | --- | --- | --- | --- | --- | --- |
| **Actinobacteria** | **Coriobacteriia** | **Coriobacteriales** | **Coriobacteriaceae** | **Collinsella** | **0.059** | **TRUE** |
| **Bacteroidetes** | **Bacteroidia** | **Bacteroidales** | **Bacteroidaceae** | **Bacteroides** | **0.029** | **TRUE** |
| **Bacteroidetes** | **Bacteroidia** | **Bacteroidales** | **Rikenellaceae** | **Alistipes** | **0.027** | **TRUE** |
| **Firmicutes** | **Clostridia** | **Clostridiales** | **Ruminococcaceae** | **Faecalibacterium** | **0.059** | **TRUE** |
| **Proteobacteria** | **Alphaproteobacteria** | **Caulobacterales** | **Caulobacteraceae** | **Brevundimonas** | **0.027** | **TRUE** |
| **Proteobacteria** | **Alphaproteobacteria** | **Sphingomonadales** | **Sphingomonadaceae** | **Novosphingobium** | **0.067** | **TRUE** |
| **Proteobacteria** | **Betaproteobacteria** | **Neisseriales** | **Neisseriaceae** | **UC_Neisseriaceae** | **0.02** | **TRUE** |
| **Proteobacteria** | **Gammaproteobacteria** | **Aeromonadales** | **Aeromonadaceae** | **Aeromonas** | **0.059** | **TRUE** |
| **Proteobacteria** | **Gammaproteobacteria** | **Enterobacterales** | **Enterobacteriaceae** | **Klebsiella** | **0.029** | **TRUE** |
| Acidobacteria | Blastocatellia | Blastocatellales | Blastocatellaceae | Blastocatella | NA | FALSE |
| Acidobacteria | Blastocatellia | Blastocatellales | Blastocatellaceae | Stenotrophobacter | NA | FALSE |
| Actinobacteria | Coriobacteriia | Coriobacteriales | Coriobacteriaceae | uc_Coriobacteriaceae_f | 0.276 | FALSE |
| Actinobacteria | Actinobacteria_c | Bifidobacteriales | Bifidobacteriaceae | Bifidobacterium | 0.320 | FALSE |
| Actinobacteria | Actinobacteria_c | Micrococcales | uc_Micrococcales_o | uc_Micrococcales_o | 0.347 | FALSE |
| Actinobacteria | Actinobacteria_c | Corynebacteriales | Nocardiaceae | Rhodococcus | 0.402 | FALSE |
| Actinobacteria | Actinobacteria_c | Micrococcales | Microbacteriaceae | Microbacterium | 0.500 | FALSE |
| Actinobacteria | Actinobacteria_c | Propionibacteriales | Propionibacteriaceae | uc_Propionibacteriaceae_f | 0.613 | FALSE |
| Actinobacteria | Actinobacteria_c | Micrococcales | Micrococcaceae | Rothia | 0.726 | FALSE |
| Actinobacteria | Actinobacteria_c | Corynebacteriales | Mycobacteriaceae | Mycobacterium | 0.787 | FALSE |
| Actinobacteria | Actinobacteria_c | Propionibacteriales | Nocardioidaceae | Nocardioides | 0.787 | FALSE |
| Actinobacteria | Actinobacteria_c | uc_Actinobacteria_c | uc_Actinobacteria_c | uc_Actinobacteria_c | 0.787 | FALSE |
| Actinobacteria | Actinobacteria_c | Corynebacteriales | Nocardiaceae | Gordonia | 0.806 | FALSE |
| Actinobacteria | Actinobacteria_c | Micrococcales | Dermabacteraceae | uc_Dermabacteraceae_f | 0.862 | FALSE |
| Actinobacteria | Actinobacteria_c | Propionibacteriales | Propionibacteriaceae | Acidipropionibacterium | 0.862 | FALSE |
| Actinobacteria | Coriobacteriia | Coriobacteriales | Coriobacteriaceae | Atopobium | 0.862 | FALSE |
| Actinobacteria | Actinobacteria_c | Micrococcales | Dermacoccaceae | uc_Dermacoccaceae_f | 0.942 | FALSE |
| Actinobacteria | Actinobacteria_c | Actinomycetales | Actinomycetaceae | Actinomyces | 0.963 | FALSE |
| Actinobacteria | Actinobacteria_c | Propionibacteriales | Propionibacteriaceae | Cutibacterium | 0.998 | FALSE |
| Actinobacteria | Actinobacteria_c | Corynebacteriales | Corynebacteriaceae | Corynebacterium | 1.000 | FALSE |
| Actinobacteria | Actinobacteria_c | Actinomycetales | Actinomycetaceae | uc_Actinomycetaceae_f | NA | FALSE |
| Actinobacteria | Actinobacteria_c | Corynebacteriales | Dietziaceae | Dietzia | NA | FALSE |
| Actinobacteria | Actinobacteria_c | Frankiales | Frankiaceae | Frankia | NA | FALSE |
| Actinobacteria | Actinobacteria_c | Frankiales | Geodermatophilaceae | Modestobacter | NA | FALSE |
| Actinobacteria | Actinobacteria_c | Frankiales | Geodermatophilaceae | uc_Geodermatophilaceae_f | NA | FALSE |
| Actinobacteria | Actinobacteria_c | Kineosporiales | Kineosporiaceae | uc_Kineosporiaceae_f | NA | FALSE |
| Actinobacteria | Actinobacteria_c | Micrococcales | Intrasporangiaceae | Ornithinimicrobium | NA | FALSE |
| Actinobacteria | Actinobacteria_c | Micrococcales | Micrococcaceae | Kocuria | NA | FALSE |
| Actinobacteria | Actinobacteria_c | Micrococcales | Micrococcaceae | Micrococcus | NA | FALSE |
| Actinobacteria | Actinobacteria_c | Propionibacteriales | Nocardioidaceae | uc_Nocardioidaceae_f | NA | FALSE |
| Actinobacteria | Actinobacteria_c | Propionibacteriales | Propionibacteriaceae | Friedmanniella | NA | FALSE |
| Actinobacteria | Actinobacteria_c | Propionibacteriales | Propionibacteriaceae | Microlunatus | NA | FALSE |
| Actinobacteria | Actinobacteria_c | Pseudonocardiales | Pseudonocardiaceae | Pseudonocardia | NA | FALSE |
| Actinobacteria | Actinobacteria_c | Pseudonocardiales | Pseudonocardiaceae | Saccharothrix | NA | FALSE |
| Actinobacteria | Actinobacteria_c | Streptomycetales | Streptomycetaceae | Streptomyces | NA | FALSE |
| Actinobacteria | Thermoleophilia | Thermoleophilales | Thermoleophilaceae | PAC000142_g | NA | FALSE |
| Armatimonadetes | Fimbriimonadia | Fimbriimonadales | Fimbriimonadaceae | Fimbriimonas | 0.726 | FALSE |
| Bacteroidetes | Flavobacteria | Flavobacteriales | Flavobacteriaceae | Cloacibacterium | 0.158 | FALSE |
| Bacteroidetes | Bacteroidia | Bacteroidales | Porphyromonadaceae | Parabacteroides | 0.347 | FALSE |
| Bacteroidetes | Flavobacteria | Flavobacteriales | Flavobacteriaceae | Flavobacterium | 0.397 | FALSE |
| Bacteroidetes | Flavobacteria | Flavobacteriales | Flavobacteriaceae | Chryseobacterium | 0.528 | FALSE |
| Bacteroidetes | Sphingobacteriia | Sphingobacteriales | Chitinophagaceae | Sediminibacterium | 0.751 | FALSE |
| Bacteroidetes | Bacteroidia | Bacteroidales | Prevotellaceae | Alloprevotella | 0.787 | FALSE |
| Bacteroidetes | Flavobacteria | Flavobacteriales | Flavobacteriaceae | Bergeyella | 0.787 | FALSE |
| Bacteroidetes | Bacteroidia | Bacteroidales | Prevotellaceae | Prevotella | 0.901 | FALSE |
| Bacteroidetes | Flavobacteria | Flavobacteriales | Flavobacteriaceae | uc_Flavobacteriaceae_f | 0.911 | FALSE |
| Bacteroidetes | Sphingobacteriia | Sphingobacteriales | Chitinophagaceae | uc_Chitinophagaceae_f | 0.931 | FALSE |
| Bacteroidetes | Bacteroidia | Bacteroidales | Porphyromonadaceae | Porphyromonas | 0.985 | FALSE |
| Bacteroidetes | Bacteroidia | Bacteroidales | Porphyromonadaceae | Dysgonomonas | NA | FALSE |
| Bacteroidetes | Bacteroidia | Bacteroidales | Porphyromonadaceae | Tannerella | NA | FALSE |
| Bacteroidetes | Cytophagia | Cytophagales | Hymenobacteraceae | Hymenobacter | NA | FALSE |
| Bacteroidetes | Flavobacteria | Flavobacteriales | uc_Flavobacteriales_o | uc_Flavobacteriales_o | NA | FALSE |
| Bacteroidetes | Sphingobacteriia | Sphingobacteriales | Chitinophagaceae | Ferruginibacter | NA | FALSE |
| Bacteroidetes | Sphingobacteriia | Sphingobacteriales | Chitinophagaceae | Terrimonas | NA | FALSE |
| Chloroflexi | uc_Chloroflexi_p | uc_Chloroflexi_p | uc_Chloroflexi_p | uc_Chloroflexi_p | NA | FALSE |
| Cyanobacteria | Chroobacteria | Oscillatoriales | uc_Oscillatoriales_o | uc_Oscillatoriales_o | 0.806 | FALSE |
| Cyanobacteria | Chroobacteria | Oscillatoriales | Prochlorotrichaceae | uc_Prochlorotrichaceae_f | 0.862 | FALSE |
| Cyanobacteria | Chroobacteria | uc_Chroobacteria_c | uc_Chroobacteria_c | uc_Chroobacteria_c | 0.911 | FALSE |
| Cyanobacteria | Hormogoneae | Nostocales | Nostocaceae | uc_Nostocaceae_f | NA | FALSE |
| Cyanobacteria | uc_Cyanobacteria_p | uc_Cyanobacteria_p | uc_Cyanobacteria_p | PAC000053_g | NA | FALSE |
| Cyanobacteria | uc_Cyanobacteria_p | uc_Cyanobacteria_p | uc_Cyanobacteria_p | AY212703_g | NA | FALSE |
| Deinococcus-Thermus | Deinococci | Deinococcales | Deinococcaceae | Deinococcus | 0.606 | FALSE |
| Deinococcus-Thermus | Deinococci | Thermales | Thermaceae | Meiothermus | 0.862 | FALSE |
| Deinococcus-Thermus | Deinococci | Thermales | Thermaceae | Thermus | NA | FALSE |
| Firmicutes | Clostridia | Clostridiales | Lachnospiraceae | Blautia | 0.136 | FALSE |
| Firmicutes | Clostridia | Clostridiales | Ruminococcaceae | uc_Ruminococcaceae_f | 0.136 | FALSE |
| Firmicutes | Clostridia | Clostridiales | Peptostreptococcaceae | uc_Peptostreptococcaceae_f | 0.158 | FALSE |
| Firmicutes | Clostridia | Clostridiales | Ruminococcaceae | Monoglobus | 0.158 | FALSE |
| Firmicutes | Erysipelotrichi | Erysipelotrichales | Erysipelotrichaceae | Longicatena | 0.158 | FALSE |
| Firmicutes | Clostridia | Clostridiales | Clostridiaceae | Clostridium | 0.276 | FALSE |
| Firmicutes | Clostridia | Clostridiales | Ruminococcaceae | Ruminococcus | 0.276 | FALSE |
| Firmicutes | Clostridia | Clostridiales | Christensenellaceae | Christensenella | 0.347 | FALSE |
| Firmicutes | Clostridia | Clostridiales | Lachnospiraceae | Clostridium_g21 | 0.347 | FALSE |
| Firmicutes | Clostridia | Clostridiales | Lachnospiraceae | PAC001201_g | 0.347 | FALSE |
| Firmicutes | Clostridia | Clostridiales | Lachnospiraceae | Syntrophococcus | 0.347 | FALSE |
| Firmicutes | Clostridia | Clostridiales | Mogibacterium_f | PAC001168_g | 0.347 | FALSE |
| Firmicutes | Clostridia | Clostridiales | Mogibacterium_f | PAC001609_g | 0.347 | FALSE |
| Firmicutes | Clostridia | Clostridiales | Peptostreptococcaceae | Romboutsia | 0.347 | FALSE |
| Firmicutes | Clostridia | Clostridiales | Ruminococcaceae | Caproiciproducens | 0.347 | FALSE |
| Firmicutes | Clostridia | Clostridiales | Ruminococcaceae | Eubacterium_g23 | 0.347 | FALSE |
| Firmicutes | Clostridia | Clostridiales | Ruminococcaceae | Oscillibacter | 0.347 | FALSE |
| Firmicutes | Clostridia | Clostridiales | Ruminococcaceae | Paludicola | 0.347 | FALSE |
| Firmicutes | Clostridia | Clostridiales | Ruminococcaceae | Pseudoflavonifractor | 0.347 | FALSE |
| Firmicutes | Clostridia | Clostridiales | Ruminococcaceae | Sporobacter | 0.347 | FALSE |
| Firmicutes | Erysipelotrichi | Erysipelotrichales | Erysipelotrichaceae | AB237727_g | 0.347 | FALSE |
| Firmicutes | Erysipelotrichi | Erysipelotrichales | Erysipelotrichaceae | Coprobacillus | 0.347 | FALSE |
| Firmicutes | Clostridia | Clostridiales | Ruminococcaceae | Subdoligranulum | 0.397 | FALSE |
| Firmicutes | Erysipelotrichi | Erysipelotrichales | Erysipelotrichaceae | Clostridium_g6 | 0.496 | FALSE |
| Firmicutes | Clostridia | Clostridiales | Eubacteriaceae | Eubacterium | 0.500 | FALSE |
| Firmicutes | Clostridia | Clostridiales | Lachnospiraceae | Clostridium_g24 | 0.500 | FALSE |
| Firmicutes | Clostridia | Clostridiales | Lachnospiraceae | uc_Lachnospiraceae_f | 0.606 | FALSE |
| Firmicutes | Clostridia | Clostridiales | Lachnospiraceae | Ruminococcus_g4 | 0.626 | FALSE |
| Firmicutes | Bacilli | Bacillales | Staphylococcaceae | Staphylococcus | 0.699 | FALSE |
| Firmicutes | Bacilli | Bacillales | Paenibacillaceae | Paenibacillus | 0.726 | FALSE |
| Firmicutes | Negativicutes | Veillonellales | Veillonellaceae | Veillonella | 0.726 | FALSE |
| Firmicutes | Bacilli | Bacillales | Staphylococcaceae | Macrococcus | 0.787 | FALSE |
| Firmicutes | Bacilli | Bacillales | uc_Bacillales_o | uc_Bacillales_o | 0.787 | FALSE |
| Firmicutes | Negativicutes | Veillonellales | Veillonellaceae | Dialister | 0.787 | FALSE |
| Firmicutes | Bacilli | Bacillales | Gemella_f | Gemella | 0.802 | FALSE |
| Firmicutes | Clostridia | Clostridiales | Lachnospiraceae | Eubacterium_g5 | 0.802 | FALSE |
| Firmicutes | Bacilli | Lactobacillales | Enterococcaceae | Enterococcus | 0.806 | FALSE |
| Firmicutes | Bacilli | Bacillales | Bacillaceae | Bacillus | 0.808 | FALSE |
| Firmicutes | Bacilli | Lactobacillales | Aerococcaceae | Granulicatella | 0.862 | FALSE |
| Firmicutes | Tissierellia | Tissierellales | Peptoniphilaceae | Peptoniphilus | 0.901 | FALSE |
| Firmicutes | Bacilli | Lactobacillales | Aerococcaceae | uc_Aerococcaceae_f | 0.911 | FALSE |
| Firmicutes | Bacilli | Lactobacillales | Streptococcaceae | Streptococcus | 0.961 | FALSE |
| Firmicutes | Tissierellia | Tissierellales | Peptoniphilaceae | uc_Peptoniphilaceae_f | 0.985 | FALSE |
| Firmicutes | Tissierellia | Tissierellales | Peptoniphilaceae | Anaerococcus | 0.998 | FALSE |
| Firmicutes | Bacilli | Bacillales | Planococcaceae | Lysinibacillus | NA | FALSE |
| Firmicutes | Bacilli | Bacillales | Planococcaceae | Planococcus | NA | FALSE |
| Firmicutes | Bacilli | Bacillales | Staphylococcaceae | Salinicoccus | NA | FALSE |
| Firmicutes | Bacilli | Bacillales | Thermoactinomycetaceae | Thermoactinomyces | NA | FALSE |
| Firmicutes | Bacilli | Lactobacillales | Aerococcaceae | Aerococcus | NA | FALSE |
| Firmicutes | Bacilli | Lactobacillales | Enterococcaceae | Vagococcus | NA | FALSE |
| Firmicutes | Bacilli | Lactobacillales | Lactobacillaceae | Lactobacillus | NA | FALSE |
| Firmicutes | Bacilli | uc_Bacilli_c | uc_Bacilli_c | uc_Bacilli_c | NA | FALSE |
| Firmicutes | Clostridia | Clostridiales | Christensenellaceae | PAC001437_g | NA | FALSE |
| Firmicutes | Clostridia | Clostridiales | Christensenellaceae | uc_Christensenellaceae_f | NA | FALSE |
| Firmicutes | Clostridia | Clostridiales | Eubacteriaceae | Anaerofustis | NA | FALSE |
| Firmicutes | Clostridia | Clostridiales | Peptococcaceae | Peptococcus | NA | FALSE |
| Firmicutes | Clostridia | Clostridiales | Peptostreptococcaceae | Peptostreptococcus | NA | FALSE |
| Firmicutes | Clostridia | Clostridiales | Ruminococcaceae | Agathobaculum | NA | FALSE |
| Firmicutes | Clostridia | Clostridiales | Ruminococcaceae | PAC000672_g | NA | FALSE |
| Firmicutes | Clostridia | Clostridiales | Ruminococcaceae | PAC001402_g | NA | FALSE |
| Firmicutes | Clostridia | Clostridiales | Ruminococcaceae | PAC001468_g | NA | FALSE |
| Firmicutes | Clostridia | Clostridiales | Ruminococcaceae | PAC002194_g | NA | FALSE |
| Firmicutes | Negativicutes | Acidaminococcales | Acidaminococcaceae | Acidaminococcus | NA | FALSE |
| Firmicutes | Negativicutes | Veillonellales | Veillonellaceae | uc_Veillonellaceae_f | NA | FALSE |
| Firmicutes | Tissierellia | Tissierellales | Tissierellaceae | Anaerosalibacter | NA | FALSE |
| Firmicutes | Tissierellia | Tissierellales | Tissierellaceae | uc_Tissierellaceae_f | NA | FALSE |
| Firmicutes | Tissierellia | Tissierellales | uc_Tissierellales_o | uc_Tissierellales_o | NA | FALSE |
| Fusobacteria | Fusobacteria_c | Fusobacteriales | Fusobacteriaceae | Fusobacterium | 0.787 | FALSE |
| Gemmatimonadetes | Longimicrobia | Longimicrobiales | GQ263002_f | GQ263002_g | NA | FALSE |
| Parcubacteria_OD1 | Paceibacter_c | uc_Paceibacter_c | uc_Paceibacter_c | uc_Paceibacter_c | NA | FALSE |
| Proteobacteria | Betaproteobacteria | Burkholderiales | Comamonadaceae | Acidovorax | 0.103 | FALSE |
| Proteobacteria | Gammaproteobacteria | Xanthomonadales | Xanthomonadaceae | uc_Xanthomonadaceae_f | 0.158 | FALSE |
| Proteobacteria | Gammaproteobacteria | Pseudomonadales | Moraxellaceae | uc_Moraxellaceae_f | 0.173 | FALSE |
| Proteobacteria | Gammaproteobacteria | Enterobacterales | Enterobacteriaceae | Citrobacter | 0.227 | FALSE |
| Proteobacteria | Gammaproteobacteria | Enterobacterales | Enterobacteriaceae | Escherichia | 0.235 | FALSE |
| Proteobacteria | Gammaproteobacteria | Enterobacterales | Enterobacteriaceae | Enterobacter | 0.255 | FALSE |
| Proteobacteria | Alphaproteobacteria | Sphingomonadales | Erythrobacteraceae | Erythrobacter | 0.276 | FALSE |
| Proteobacteria | Gammaproteobacteria | Enterobacterales | Enterobacteriaceae | uc_Enterobacteriaceae_f | 0.321 | FALSE |
| Proteobacteria | Gammaproteobacteria | Xanthomonadales | Xanthomonadaceae | Luteimonas | 0.347 | FALSE |
| Proteobacteria | Gammaproteobacteria | Xanthomonadales | Xanthomonadaceae | Stenotrophomonas | 0.372 | FALSE |
| Proteobacteria | Oligoflexia | Bdellovibrionales | AB511016_f | AB511016_g | 0.397 | FALSE |
| Proteobacteria | Betaproteobacteria | Burkholderiales | Comamonadaceae | Comamonas | 0.425 | FALSE |
| Proteobacteria | Alphaproteobacteria | Rhizobiales | Bradyrhizobiaceae | Bradyrhizobium | 0.444 | FALSE |
| Proteobacteria | Gammaproteobacteria | Xanthomonadales | Xanthomonadaceae | Xanthomonas | 0.500 | FALSE |
| Proteobacteria | Alphaproteobacteria | Rhizobiales | uc_Rhizobiales_o | uc_Rhizobiales_o | 0.500 | FALSE |
| Proteobacteria | Alphaproteobacteria | Sphingomonadales | Sphingomonadaceae | Sphingomonas | 0.500 | FALSE |
| Proteobacteria | Gammaproteobacteria | Pseudomonadales | Pseudomonadaceae | Pseudomonas | 0.500 | FALSE |
| Proteobacteria | Alphaproteobacteria | Rhizobiales | Methylobacteriaceae | Methylobacterium | 0.511 | FALSE |
| Proteobacteria | Alphaproteobacteria | Caulobacterales | Caulobacteraceae | Caulobacter | 0.606 | FALSE |
| Proteobacteria | Betaproteobacteria | Burkholderiales | Burkholderiaceae | Burkholderia | 0.608 | FALSE |
| Proteobacteria | Alphaproteobacteria | Caulobacterales | Caulobacteraceae | uc_Caulobacteraceae_f | 0.626 | FALSE |
| Proteobacteria | Betaproteobacteria | Burkholderiales | Oxalobacteraceae | Janthinobacterium | 0.626 | FALSE |
| Proteobacteria | Betaproteobacteria | Burkholderiales | Alcaligenaceae | Alcaligenes | 0.700 | FALSE |
| Proteobacteria | Gammaproteobacteria | Enterobacterales | Yersiniaceae | Serratia | 0.771 | FALSE |
| Proteobacteria | Gammaproteobacteria | Pasteurellales | Pasteurellaceae | Haemophilus | 0.771 | FALSE |
| Proteobacteria | Alphaproteobacteria | Rhodobacterales | Rhodobacteraceae | Paracoccus | 0.772 | FALSE |
| Proteobacteria | Alphaproteobacteria | Rhizobiales | Methylobacteriaceae | uc_Methylobacteriaceae_f | 0.787 | FALSE |
| Proteobacteria | Alphaproteobacteria | Rhizobiales | Rhizobiaceae | Rhizobium | 0.787 | FALSE |
| Proteobacteria | Betaproteobacteria | Burkholderiales | Alcaligenaceae | Achromobacter | 0.787 | FALSE |
| Proteobacteria | Deltaproteobacteria | Myxococcales | Polyangiaceae | Labilithrix | 0.787 | FALSE |
| Proteobacteria | Deltaproteobacteria | PAC002565_o | EU861868_f | EU861868_g | 0.787 | FALSE |
| Proteobacteria | Epsilonproteobacteria | Campylobacterales | Campylobacteraceae | Campylobacter | 0.787 | FALSE |
| Proteobacteria | Gammaproteobacteria | Enterobacterales | Morganellaceae | uc_Morganellaceae_f | 0.802 | FALSE |
| Proteobacteria | Gammaproteobacteria | Pasteurellales | Pasteurellaceae | uc_Pasteurellaceae_f | 0.802 | FALSE |
| Proteobacteria | Betaproteobacteria | Burkholderiales | Comamonadaceae | Delftia | 0.900 | FALSE |
| Proteobacteria | Betaproteobacteria | Neisseriales | Neisseriaceae | Neisseria | 0.906 | FALSE |
| Proteobacteria | Gammaproteobacteria | Alteromonadales | Shewanellaceae | Shewanella | 0.911 | FALSE |
| Proteobacteria | Betaproteobacteria | Burkholderiales | Oxalobacteraceae | Massilia | 0.942 | FALSE |
| Proteobacteria | Betaproteobacteria | Burkholderiales | Comamonadaceae | uc_Comamonadaceae_f | 0.967 | FALSE |
| Proteobacteria | Gammaproteobacteria | Pseudomonadales | Moraxellaceae | Acinetobacter | 0.988 | FALSE |
| Proteobacteria | Alphaproteobacteria | Rhizobiales | Bosea_f | Bosea | NA | FALSE |
| Proteobacteria | Alphaproteobacteria | Rhizobiales | Hyphomicrobiaceae | Pedomicrobium | NA | FALSE |
| Proteobacteria | Alphaproteobacteria | Rhizobiales | Methylobacteriaceae | Microvirga | NA | FALSE |
| Proteobacteria | Alphaproteobacteria | Rhodobacterales | Rhodobacteraceae | Rubellimicrobium | NA | FALSE |
| Proteobacteria | Alphaproteobacteria | Rhodobacterales | Rhodobacteraceae | uc_Rhodobacteraceae_f | NA | FALSE |
| Proteobacteria | Alphaproteobacteria | Rhodospirillales | Acetobacteraceae | Gluconacetobacter | NA | FALSE |
| Proteobacteria | Alphaproteobacteria | Rhodospirillales | uc_Rhodospirillales_o | uc_Rhodospirillales_o | NA | FALSE |
| Proteobacteria | Alphaproteobacteria | Rhodospirillales | Rhodospirillaceae | Skermanella | NA | FALSE |
| Proteobacteria | Alphaproteobacteria | Sphingomonadales | Erythrobacteraceae | Porphyrobacter | NA | FALSE |
| Proteobacteria | Alphaproteobacteria | Sphingomonadales | Erythrobacteraceae | uc_Erythrobacteraceae_f | NA | FALSE |
| Proteobacteria | Alphaproteobacteria | Sphingomonadales | Sphingomonadaceae | Sphingobium | NA | FALSE |
| Proteobacteria | Alphaproteobacteria | Sphingomonadales | Sphingomonadaceae | Sphingopyxis | NA | FALSE |
| Proteobacteria | Alphaproteobacteria | Sphingomonadales | Sphingomonadaceae | uc_Sphingomonadaceae_f | NA | FALSE |
| Proteobacteria | Alphaproteobacteria | uc_Alphaproteobacteria_c | uc_Alphaproteobacteria_c | uc_Alphaproteobacteria_c | NA | FALSE |
| Proteobacteria | Betaproteobacteria | Burkholderiales | Comamonadaceae | Curvibacter | NA | FALSE |
| Proteobacteria | Betaproteobacteria | Burkholderiales | Oxalobacteraceae | uc_Oxalobacteraceae_f | NA | FALSE |
| Proteobacteria | Betaproteobacteria | Burkholderiales | Ralstonia_f | Cupriavidus | NA | FALSE |
| Proteobacteria | Epsilonproteobacteria | Campylobacterales | Sulfurovum_f | Sulfurovum | NA | FALSE |
| Proteobacteria | Gammaproteobacteria | Cellvibrionales | Cellvibrionaceae | Cellvibrio | NA | FALSE |
| Proteobacteria | Gammaproteobacteria | Pasteurellales | Pasteurellaceae | Aggregatibacter | NA | FALSE |
| Proteobacteria | Gammaproteobacteria | Pseudomonadales | Moraxellaceae | Moraxella | NA | FALSE |
| Proteobacteria | Gammaproteobacteria | uc_Gammaproteobacteria1_c | uc_Gammaproteobacteria1_c | uc_Gammaproteobacteria1_c | NA | FALSE |
| Proteobacteria | Gammaproteobacteria | uc_Gammaproteobacteria2_c | uc_Gammaproteobacteria2_c | uc_Gammaproteobacteria2_c | NA | FALSE |
| Proteobacteria | Oligoflexia | Bdellovibrionales | Bdellovibrionaceae | Bdellovibrio | NA | FALSE |
| Proteobacteria | Oligoflexia | Oligoflexales | Oligoflexaceae | PAC000680_g | NA | FALSE |
| Saccharibacteria_TM7 | Saccharimonas_c | Saccharimonas_o | Saccharimonas_f | Saccharimonas | 0.787 | FALSE |
| Spirochaetes | Spirochaetia | Spirochaetales | Spirochaetaceae | uc_Spirochaetaceae_f | NA | FALSE |
| Synergistetes | Synergistia | Synergistales | Synergistaceae | uc_Synergistaceae_f | NA | FALSE |
| Verrucomicrobia | Verrucomicrobiae | Verrucomicrobiales | Akkermansiaceae | Akkermansia | 0.420 | FALSE |
| Verrucomicrobia | Verrucomicrobiae | Verrucomicrobiales | Akkermansiaceae | HM630201_g | NA | FALSE |
| NA, not analyzed |  |  |  |  |  |  |

**Supplementary Table S2.** Correlation between covariates and microbiota variation. Correlations were determined using EnvFit model based on the Bray-Curtis dissimilarity of microbiota.

| Clinicopathological factors | R squared value | *P*-value |
| --- | --- | --- |
| Tissue type (benign vs. malignant) | 0.036 | **0.042** |
| IPSS-Q1 | 0.064 | 0.059 |
| Positive surgical margin (focal) | 0.058 | 0.062 |
| Tumor location (lobe) on MRI | 0.062 | 0.074 |
| IPSS-Q7 | 0.054 | 0.093 |
| PSA (Prostate-specific antigen) | 0.05 | 0.107 |
| Tumor length per core | 0.045 | 0.127 |
| Tumor percentage per core | 0.042 | 0.131 |
| Positive surgical margin | 0.042 | 0.134 |
| Number of biopsy cores positive for cancer | 0.041 | 0.148 |
| DM (Diabetes Mellitus) | 0.041 | 0.156 |
| Smoking amount (pack-year) | 0.039 | 0.161 |
| Mean IIEF-5 | 0.04 | 0.177 |
| Percentage of biopsy cores positive for cancer | 0.035 | 0.196 |
| Mean tumor volume | 0.034 | 0.214 |
| Location of positive surgical margin | 0.017 | 0.223 |
| Length of hospital stay | 0.035 | 0.228 |
| IPSS-Q4 | 0.035 | 0.229 |
| Mean IPSS | 0.032 | 0.24 |
| Pathologic stage | 0.031 | 0.254 |
| Lymph node enlargement on MRI | 0.03 | 0.268 |
| Tumor location (zone) on MRI | 0.029 | 0.272 |
| 1st sum GS | 0.029 | 0.288 |
| Hormone therapy | 0.025 | 0.299 |
| Biochemical recurrence | 0.024 | 0.311 |
| Biopsy | 0.026 | 0.325 |
| Secondary pathologic GS | 0.027 | 0.335 |
| Membranous urethral length (mm) on MRI | 0.024 | 0.351 |
| Neoadjuvant hormone therapy | 0.021 | 0.357 |
| Operative time | 0.024 | 0.359 |
| Bladder neck invasion | 0.021 | 0.362 |
| Pathologic Gleason score | 0.024 | 0.373 |
| Prostate Imaging Reporting & Data System (PIRADS) score | 0.022 | 0.375 |
| Tertiary pathologic GS | 0.019 | 0.386 |
| Prostate volume on transrectal ultrasound (TRUS) | 0.021 | 0.399 |
| Length of Foley catheter indwelling | 0.018 | 0.43 |
| IPSS-Q3 | 0.02 | 0.431 |
| Height | 0.02 | 0.442 |
| Number of boipsy core | 0.016 | 0.448 |
| Extracapsular extension | 0.019 | 0.456 |
| National Comprehensive Cancer Network | 0.018 | 0.477 |
| Smoking history | 0.016 | 0.498 |
| Biopsy core length | 0.016 | 0.5 |
| IPSS-Q6 | 0.015 | 0.526 |
| Prostatic intraepithelial neoplasia | 0.013 | 0.528 |
| Digital rectal examination | 0.014 | 0.563 |
| Multicentricity | 0.013 | 0.573 |
| Venous invasion | 0.01 | 0.575 |
| Number of PIRADS≥4 lesion on MRI | 0.013 | 0.583 |
| IPSS-Q5 | 0.012 | 0.601 |
| Body mass index | 0.011 | 0.615 |
| Clinical stage | 0.011 | 0.63 |
| Family history | 0.008 | 0.652 |
| Complication | 0.008 | 0.66 |
| Seminal vesicle invasion | 0.01 | 0.664 |
| PSA density | 0.008 | 0.676 |
| Pathologic prostate volume | 0.004 | 0.677 |
| Weight | 0.008 | 0.709 |
| IPSS-QOL | 0.007 | 0.736 |
| Primary biopsy GS | 0.006 | 0.756 |
| Console time | 0.006 | 0.77 |
| HTN | 0.005 | 0.803 |
| Primary pathologic GS | 0.004 | 0.852 |
| Co-Morbidity | 0.002 | 0.895 |
| Clinical stage | 0.003 | 0.899 |
| Age at surgery | 0.002 | 0.908 |
| 1st p-Localization | 0.002 | 0.911 |
| Biopsy Gleason score | 0.002 | 0.918 |
| Perineural invasion | 0.002 | 0.918 |
| IPSS-Q2 | 0.002 | 0.924 |
| Angiolymphatic invasion | 0.002 | 0.925 |
| Secondary biopsy GS | 0.002 | 0.928 |
| Blood transfusion | 0 | 1 |
| Radiothearpy | 0 | 1 |

**Supplementary Table S3.** Comparison of clinicopathological characteristics between non-DM and DM groups. DM, diabetes mellitus.

| Characteristics | Non-DM | DM | *P*-value |
| --- | --- | --- | --- |
| Total number | 39 | 21 |  |
| Age (years) | 65.6 ± 8.4 | 68.8 ± 5.7 | 0.80 |
| Height (cm) | 167.4 ± 5.0 | 167.0 ± 6.1 | 1.00 |
| Weight (kg) | 68.2 ± 9.6 | 73.2 ± 6.8 | 0.67 |
| BMI (kg/m^2^) | 24.4 ± 3.4 | 26.3 ± 2.4 | 0.78 |
| Prostate-specific antigen (PSA) |  |  |  |
| low (< 10 ng/mL) (n, %) | 20 (51.3%) | 12 (57.1%) | 0.16 |
| intermediate (10 - 20 ng/mL) (n, %) | 7 (17.9%) | 4 (19.0%) | 0.37 |
| high (> 20 ng/mL) (n, %) | 12 (30.8%) | 5 (23.8%) | 0.09 |
| Gleason score (GS) | |  |  |
| low (< 7) (n, %) | 25 (64.1%) | 16 (76.2%) | 0.16 |
| intermediate (= 7) (n, %) | 1 (2.6%) | 2 (9.5%) | 0.56 |
| high (> 7) (n, %) | 13 (33.3%) | 3 (14.3%) | **0.01** |
| Pathologic stage | |  |  |
| low (T1-T2a) (n, %) | 1 (2.6%) | 1 (4.8%) | 1.00 |
| intermediate (T2b-T2c) (n, %) | 16 (41.0%) | 8 (38.1%) | 0.10 |
| high (> T3a) (n, %) | 22 (56.4%) | 12 (57.1%) | 0.09 |
| Multicentricity | |  |  |
| No (n, %) | 13 (33.3%) | 3 (14.3%) | **0.01** |
| Yes (n, %) | 26 (66.7%) | 18 (85.7%) | 0.23 |


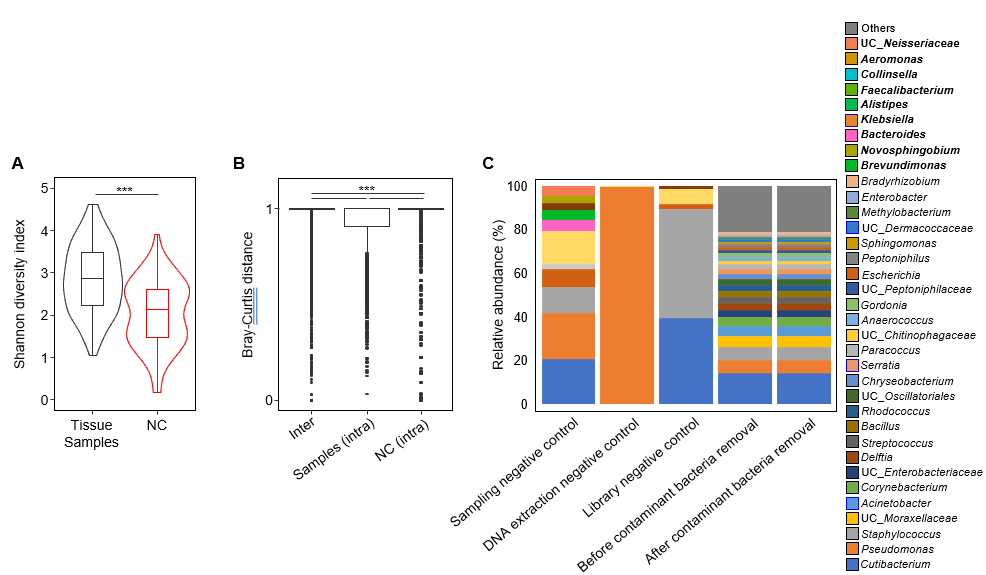


**Supplementary Figure S1.** Microbiota in samples and negative controls. (A) Shannon diversity indices of microbiota were compared between tissue samples and negative controls. (B) Intra-variations and inter-variations of microbiota in tissue samples and negative controls were compared based on the Bray-Curtis distance. (C) The mean relative abundances of detected sequences in each group. Nine genera were identified as contaminants and removed from data in tissue samples (details in Supplementary Table S1). NC, negative control. ****p* < 0.001.


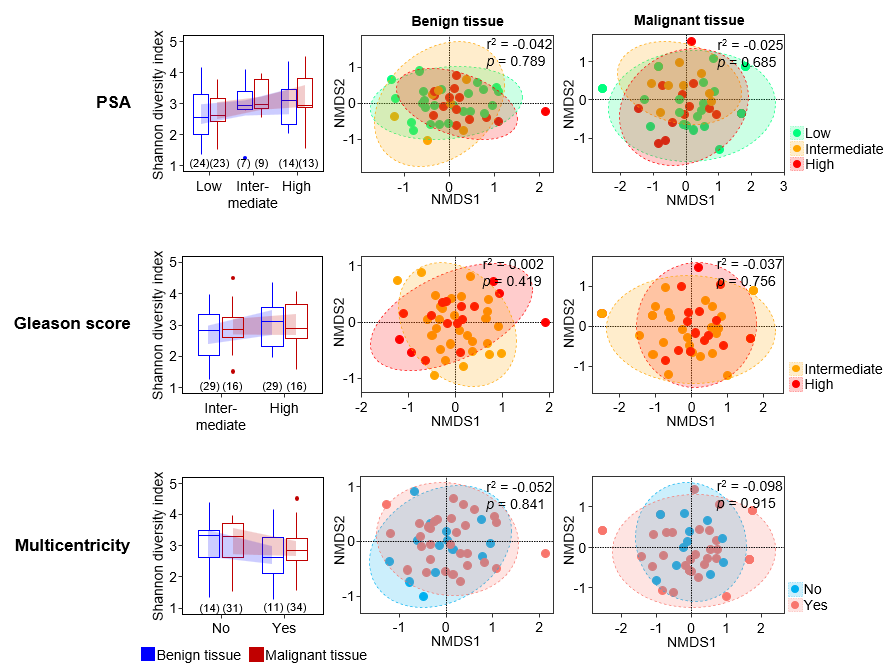


**Supplementary Figure S2.** Microbiota in each tissue were compared according to aggressiveness such as PSA, Geason score, and multicentricity. Shannon diversity indices of microbiota in each group were compared using boxplots. The difference of microbiota was analyzed in NMDS plots based on the Bray-Curtis distance. The significance in NMDS plots was calculated by ANOSIM. The numbers in brackets indicate the number of subjects in each group. PSA, prostate-specific antigen; NMDS, non-metric multidimensional scaling; ANOSIM, analysis of similarities.


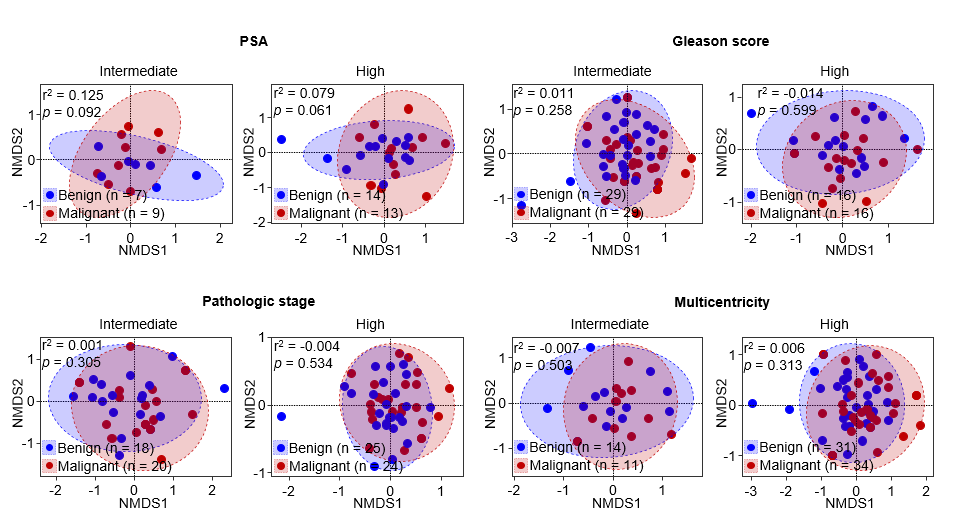


**Supplementary Figure S3.** Comparison of microbiota in each aggressiveness group between benign and malignant tissues. The difference of microbiota was analyzed in NMDS plots based on the Bray-Curtis distance. The significance in NMDS plots was calculated by ANOSIM. PSA, prostate-specific antigen; NMDS, non-metric multidimensional scaling; ANOSIM, analysis of similarities.


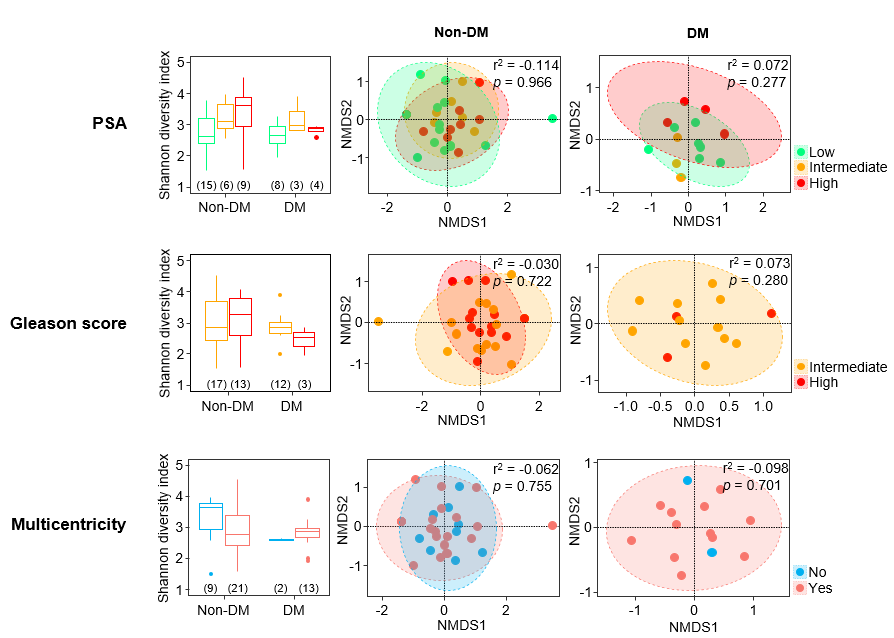


**Supplementary Figure S4.** Microbiota variations according to aggressiveness such as PSA, Geason score, and multicentricity in patients without DM and with DM. Shannon diversity index of microbiota in each group was compared using boxplots. The difference of microbiota was analyzed in NMDS plots based on the Bray-Curtis distance. The significance in NMDS plots was calculated by ANOSIM. The numbers in brackets indicate the number of subjects in each group. DM, diabetes mellitus; PSA, prostate-specific antigen; NMDS, non-metric multidimensional scaling; ANOSIM, analysis of similarities.
